# A Prospective Study of Electromyographic Amplitude Changes During Intraoperative Neural Monitoring for Open Thyroidectomy

**DOI:** 10.1007/s00268-023-07000-w

**Published:** 2023-04-01

**Authors:** Tony Lian, David Leong, Karl Ng, Sonya Bajenov, Mark Sywak

**Affiliations:** 1 Northern Clinical School, Sydney Medical School, Faculty of Medicine and Health, University of Sydney, Sydney, NSW 2006 Australia; 2 Department of Endocrine Surgery, Royal North Shore Hospital, Northern Sydney Local Health District, St Leonards, NSW 2065 Australia; 3 Department of Neurology, Royal North Shore Hospital, Northern Sydney Local Health District, St Leonards, NSW 2065 Australia; 4 Department of Anaesthesia and Pain Management, Royal North Shore Hospital, Northern Sydney Local Health District, St Leonards, NSW 2065 Australia

## Abstract

**Background:**

Intraoperative nerve monitoring (IONM) of the vagus and recurrent laryngeal nerve (RLN) enables prediction of postoperative nerve function. The underlying mechanism for loss of signal (LOS) in a visually intact nerve is poorly understood. The correlation of intraoperative electromyographic amplitude changes (EMG) with surgical manoeuvres could help identify mechanisms of LOS during conventional thyroidectomy.

**Methods:**

A prospective study of consecutive patients undergoing thyroidectomy was performed with intermittent IONM using the NIM Vital nerve monitoring system. The ipsilateral vagus and RLN was stimulated, and vagus nerve signal amplitude recorded at five time points during thyroidectomy (baseline, after mobilisation of superior pole, medialisation of the thyroid lobe, before release at Ligament of Berry, end of case). RLN signal amplitude was recorded at two time points; after medialisation of the thyroid lobe (R1), and end of case (R2).

**Results:**

A total of 100 consecutive patients undergoing thyroidectomy were studied with 126 RLN at risk. The overall rate of LOS was 4.0%. Cases without LOS demonstrated a highly significant vagus nerve median percentage amplitude drop at medialisation of the thyroid lobe (− 17.9 ± 53.1%, *P* < 0.001), and end of case (− 16.0 ± 47.2%, *P* < 0.001) compared to baseline. RLN had no significant amplitude drop at R2 compared to R1 (*P* = 0.207).

**Conclusions:**

A significant reduction in vagus nerve EMG amplitude at medialisation of the thyroid and the end of case compared to baseline indicates that stretch injury or traction forces during thyroid mobilisation are the most likely mechanism of RLN impairment during conventional thyroidectomy.

## Introduction

Routine intraoperative nerve monitoring (IONM) is widely used internationally in thyroid surgery; enabling prediction of postoperative nerve function and avoiding bilateral recurrent laryngeal nerve (RLN) palsy [1]. IONM is an adjunct to the gold standard direct visualisation and preservation of the RLN. RLN palsy, a serious complication of surgery, can cause dysphonia, dysphagia and potential airway obstruction [2]. Loss of signal (LOS), when detected with IONM, represents neurophysiological changes to the anatomically intact nerve and is a predictor for postoperative vocal cord dysfunction, allowing reconsideration of the surgical approach to staged thyroidectomy and avoiding bilateral nerve palsy [3, 4]. Type 1 LOS is defined as segmental injury, where electromyography (EMG) signal is preserved distal to the point of injury, whereas Type 2 LOS is a global injury where EMG signal is not detectable at the vagus and at the recurrent laryngeal nerve in its extralaryngeal portion, however, the intralaryngeal portion of the RLN cannot be tested [5]. INMSG guidelines have utilised an EMG cut-off of 100 μV or less to define LOS [6]. Division and permanent injury of the RLN is uncommon in experienced hands; however, LOS in a visually intact nerve occurs in up to 2–15% of thyroidectomy procedures [7–9]. The underlying mechanism for LOS in a visually intact nerve is poorly understood [8, 9]. It is postulated that a visually intact RLN with LOS intraoperatively could be a result of traction, compression at the intralaryngeal portion, or thermal injury [10].

The evaluation of IONM amplitude changes may help to identify mechanisms of LOS during conventional thyroidectomy. The aim was to conduct a prospective study using nerve integrity monitoring to measure IONM amplitude changes associated with surgical manoeuvres in patients undergoing conventional thyroidectomy and to better understand the pathophysiological changes to RLN function during thyroidectomy.

## Material and methods

A prospective study of 100 consecutive patients undergoing conventional thyroidectomy cases was performed with IONM using the NIM Vital nerve monitoring system (Medtronic TM) and TriVantage endotracheal EMG tube. This was conducted by a single surgeon and anaesthetist with a standardised surgical approach for every patient. Patients were excluded if there was RLN palsy on preoperative laryngoscopy, < 18 years of age or re-operative thyroidectomy procedures. During intubation, the anaesthetist confirmed correct endotracheal tube positioning under direct vision. The event stimulus threshold was set at 100 uV, and electrode stimulation set at 1 mA. 200 ug of Sugammadex was administered intravenously in all subjects after intubation at least 10 min prior to commencement of surgery, to reverse neuromuscular blockade and prevent confounding due to paralysis. The ipsilateral vagus nerve was stimulated, and vagus nerve signal amplitude recorded at five time points during thyroidectomy (baseline, after mobilisation of superior pole, medialisation of the thyroid lobe, before release at Ligament of Berry, and at end of case).

Five manoeuvres were completed sequentially for each thyroid lobe, with the vagus nerve stimulated following each manoeuvre. After identification and stimulation of the ipsilateral vagus nerve as a baseline reading (V1), the thyroid isthmus was divided. Subsequent to this, the superior pole was mobilised and another vagus nerve reading performed (superior pole mobilised). The inferior pole of the thyroid was then mobilised. Following this, anteromedial rotation of the gland and another vagus nerve reading was performed (medialisation). The RLN was then identified and stimulated within 2 cm of the Ligament of Berry (R1). After complete dissection of the RLN, just prior to release at the Ligament of Berry, another vagus nerve reading was performed (before release of RLN). Following removal of the thyroid gland, the vagus nerve and RLN were stimulated with the larynx in its anatomical location to mark end of case (V2) and (R2), respectively. Stimulation of the RLN was within 2 cm of the Ligament of Berry. Stimulation of the vagus nerve was conducted distal to the superior laryngeal nerve, at the level of the cricoid cartilage within the carotid sheath. In all cases of type 2 loss of signal, the surgeon waited for twenty minutes to observe for any intraoperative nerve recovery. A deidentified prospective database was maintained on the studied patients. All patients had preoperative and postoperative fibreoptic laryngoscopy to assess vocal cord function.

### Statistical analysis

Statistical analysis was conducted using GraphPad Prism 9 (Version 9.3.1). Descriptive statistics are described using mean and standard deviation or median and interquartile range depending on distribution. Wilcoxon sign rank testing was performed on electromyography absolute amplitudes and relative percentage drop from baseline. Kruskal–Wallis test and post hoc Dunn’s multiple comparisons tests were used to compare multiple categories. Logistic univariable and multivariable regression was performed to assess for patient or surgical predictors of LOS cases.

Statistical power calculations were conducted on Stata (Version 17.0). Cohen’s effect size estimates demonstrated that if the true difference in the mean response of matched pairs is 0.2, to achieve adequate statistical power of 90% with a type I error probability of 0.05, a sample size of only 26 pairs of patients is required, indicating the study was adequately powered.

## Results

A total of 100 consecutive patients undergoing thyroidectomy (26 total thyroidectomy, 74 hemithyroidectomy) were studied with 126 RLN at risk. Patient factors and surgical characteristics are summarised in Table 1. The mean patient age was 49, and the median lobe volume was 20.0 ml (Table 1). The overall rate of loss of signal (LOS) was 4.0%, with no permanent RLN injuries. There were 2 nerves with type 1 LOS, 3 nerves with type 2 LOS and 121 functionally intact nerves. All patients with intraoperative LOS were confirmed to have vocal cord palsy postoperatively which resolved in all patients at a median of 30 days post-surgery. In all patients, there was appropriate electrode placement, normal preoperative laryngoscopy and successful intraoperative identification and stimulation of the vagus nerve.

**Table 1:** Descriptive data for 100 consecutive patients with 126 nerves at risk

Variables	(n = 100)
Age (years)^a^	49 ± 17
BMI (kg/m^2^)^a^	25.4 ± 6.3
Lobe volume (ml)^b^	20.0 ± 21.6
Largest nodule size (mm)^b^	25.0 ± 19.7
Duration of surgery (mins)^b^	54 ± 14
Global LOS	5
*Indication for surgery*	
Completion thyroidectomy	10
Graves’ disease	6
Thyroiditis	6
Malignancy or risk of malignancy	17
Single nodule	34
Multinodular goitre	27

^a^Mean ± SD

^b^Median ± IQR

### Electromyography (EMG) amplitude measurements

There was a highly significant drop in vagus nerve electromyography amplitude at medialisation of the thyroid lobe (662 ± 542 mA, *P* < 0.001) and before release at the Ligament of Berry (683 ± 581 mA, *P* < 0.001) compared to baseline (810 ± 488 mA). There was also significant reduction compared to baseline at the conclusion of the procedure once all traction had been released (658 ± 578 mA, *P* < 0.001) (Table 2). There was also a highly significant vagus nerve relative percentage amplitude drop at medialisation of the thyroid lobe (− 22.1 ± 54.5%, *P* < 0.001), before release at the Ligament of Berry (− 29.8 ± 67.1%, *P* < 0.001) and at the end of case (− 17.7 ± 51.8%, *P* < 0.001) compared to baseline (Table 2). Furthermore, in the group without type 1 or type 2 LOS, there was a highly significant vagus nerve percentage amplitude drop at medialisation of the thyroid lobe (− 17.9 ± 53.1%, *P* < 0.001), before release at the Ligament of Berry (− 20.7 ± 61.7%, *P* < 0.001) and at the end of case (− 16.0 ± 47.2%, *P* < 0.001) compared to baseline. All cases of type 2 LOS were associated with a preceding 28.5% median amplitude drop from baseline and occurred exclusively during medialisation of the thyroid lobe. There were no cases of LOS which recovered intraoperatively.

**Table 2:** Median electromyography amplitudes and percentage changes from baseline of IONM vagus nerve stimulation between time points (superior pole, medialisation, before RLN release and at end of procedure) during thyroidectomy (n = 100)

Time points during surgery	EMG vagus amplitude median (IQR) (mA)	95% CI median amplitude	*P*^a^	EMG median percentage change from baseline (IQR) (%)	95% CI median amplitude percentage change	*P*^b^
Baseline (V1)	810 (488)	(701, 900)	Ref	Ref	Ref	Ref
Superior pole mobilised	774 (511)	(650, 820)	0.011	− 5.2 (27.6)	(− 10.21, 0.00)	0.015
Medialisation	662 (542)	(575, 780)	< 0.001	− 22.1 (54.5)	(− 30.93, − 13.92)	< 0.001
Before release of RLN at Ligament of Berry	683 (581)	(564, 804)	< 0.001	− 29.8 (67.1)	(− 38.10, − 18.21)	< 0.001
End of procedure (V2)	658 (578)	(562, 760)	< 0.001	− 17.7 (51.8)	(− 27.94, − 9.41)	< 0.001

^a^*P* value of median electromyography change from baseline

^b^*P* value of median electromyography percentage change from baseline

There was no significant difference between initial baseline (V1) vagus nerve amplitude recording between right (*M* = 811 ± 338 mA) and left thyroid lobes (*M* = 934 ± 575 mA) (*P* = 0.3942) in patients undergoing total thyroidectomy. Similarly, there was no significant difference in amplitude measurements on stimulation of the recurrent laryngeal nerve between R1 (*M* = 1007 ± 623 mA) and R2 in all patients (*M* = 1061 ± 589 mA, *P* = 0.229) (Table 3). Furthermore, in the group without type 1 or type 2 LOS, there was no significant difference between R1 (*M* = 1042 ± 609 mA) and R2 (*M* = 1077 ± 567 mA, *P* = 0.207) on stimulation of the recurrent laryngeal nerve.

**Table 3:** Median electromyography amplitudes of IONM recurrent laryngeal nerve stimulation after medialisation (R1) and after conclusion of the case (R2) during thyroidectomy (n = 100)

Time points during surgery	EMG amplitude median (IQR)	95% CI
R1	919 (884)	(670, 1100)
R2	979 (742)	(860, 1100)

There was a significant difference in vagus nerve EMG median amplitude compared to baseline within the four remaining timepoints (after mobilisation of superior pole, medialisation of the thyroid lobe, before release at Ligament of Berry, end of case) when analysed together with a Kruskal–Wallis test (*H* = 19.83, *P* < 0.001). However, Dunn’s post hoc multiple comparisons tests demonstrated that the vagus nerve mean EMG difference of mobilisation of the superior pole compared to baseline was not associated with a significant adjusted P value (*M* = 59.31 mA, *P* = 0.5449). Dunn’s post hoc multiple comparisons tests still demonstrated a highly significant mean difference in vagus nerve electromyography amplitude compared to baseline at medialisation of the thyroid lobe (*M* = 183.3 mA, *P* = 0.0017), before release at the Ligament of Berry (*M* = 201.3 mA, *P* < 0.001) and at end of procedure (*M* = 170.5 mA, *P* < 0.001 (Table 4).

**Table 4:** Dunn’s multiple comparisons test

Time points during surgery	Mean difference	95% CI of difference	*P*
Baseline (V1) versus superior pole mobilised	59.31	(− 31.24, 149.9)	0.5449
Baseline (V1) versus medialisation	183.3	(43.30, 323.4)	0.0017
Baseline (V1) versus before release of RLN at Ligament of Berry	201.3	(57.09, 345.6)	0.0006
Baseline (V1) versus end of procedure (V2)	170.5	(47.02, 294.1)	0.0007

### Factors predicting LOS

When univariable logistic regression was performed, patient age, sex, BMI, preoperative ultrasound largest nodule, lobe volume, central lymph node dissection or indication for surgery were not significantly associated for LOS (Table 5).

**Table 5:** Univariate logistic regression of patient variables for loss of signal

Variables	Univariate OR (95% CI)	*P*
Age (years)	0.95 (0.81, 1.11)	0.528
Sex	0.99 (0.87, 1.13)	0.579
BMI (kg/m^2^)	1.17 (0.83, 1.65)	0.374
Preoperative ultrasound largest nodule (mm)^a^	0.95 (0.82, 1.10)	0.525
Lobe volume (ml)	0.92 (0.67, 1.28)	0.640
Central lymph node dissection	0.95 (0.86, 1.20)	0.487
*Indication for surgery*		
Single/Multinodular goitre	2.54 (0.15, 31.3)	0.288
Malignancy	3.62 (0.20, 64.5)	0.381
Graves/Thyroiditis	7.25 (0.37, 140.0)	0.190

^a^log transformed

## Discussion

Intraoperative nerve monitoring with EMG provides vital information regarding the neurophysiology of RLN function and provides insight into mechanisms of temporary nerve injury [11]. Despite the gold standard visual identification of the RLN during thyroid surgery, the actual cause of transient RLN palsy is not well understood [12]. Porcine studies have demonstrated that RLN mechanical traction, compression at defined pressures, and thermal injuries caused average decreases in signal amplitude between 40 and 60% by weakening RLN conduction velocity and stimulus quality [13]. Compression of the nerve causes neuropraxia and local myelin sheath deformation in animal models and also decreases nerve conduction amplitude [14, 15]. LOS is therefore observed in all three stress mechanisms of traction, compression and thermal injury [13].

This study suggests one mechanism for LOS during thyroidectomy is related to stretch injury or traction forces during thyroid mobilisation, between the stimulation points of vagus nerve and RLN. In this study, a key finding is that in the group without type 1 or type 2 LOS, there was a highly significant vagus nerve percentage amplitude drop at medialisation of the thyroid lobe (− 17.9 ± 53.1%, *P* < 0.001), before release at the Ligament of Berry (− 20.7 ± 61.7%, *P* < 0.001) and at the end of case (− 16.0 ± 47.2%, *P* < 0.001) compared to baseline. LOS was associated with a preceding 28.5% median amplitude drop and occurred exclusively during medialisation of the thyroid lobe. Stimulation of the RLN after clear anteromedial rotation of the thyroid lobe (R1) and at the conclusion of the case (R2) was not significantly different (*P* = 0.207). In our two cases with Type 1 LOS, no signal was present in V2 but an intact RLN signal present. This highlights the importance of testing V2 at the end of the procedure rather than relying on an intact RLN signal alone.

It has been suggested that mechanisms that could result in LOS include excessive stretching of the distal RLN at the Ligament of Berry, which can cause injury to the fixed end of the nerve and its curve around the aortic arch and subclavian artery [12, 16]. Another postulated mechanism is traction on the extralaryngeal segment of the RLN during thyroid mobilisation, for example by pulling the RLN forward into an artificial genu above the inferior thyroid artery, out of the tracheoesophageal groove, which stresses the nerve [17]. Furthermore, RLN stretching from anteromedial rotation of the thyroid has been documented to increase nerve diameter, which is a possible mechanism for neuropraxia and hence, IONM amplitude reduction [18, 19].

Reductions in EMG amplitude suggest neurophysiological changes such as impaired propagation of action potentials along the nerve or diminished synaptic transmission [20]. Traction injury to the RLN is associated with histopathological morphology changes in the epineurium and perineurium of the nerve, including reductions in myelin thickness and axonal integrity [16, 21]. Amplitude decreases signify less nerve fibres participating in response to neural stimulation, and combined events of amplitude decreases and latency increases are strongly associated with LOS and RLN palsy [22]. In contrast, EMG amplitude in thyroidectomy often recovers when traction on the nerve is relieved [20, 23, 24].

Another distinctive finding in this study was that in type 2 LOS, all cases occurred after anteromedial rotation of the thyroid lobe. In the three cases with Type 2 LOS, there was no signal evident at testing of the RLN up until the point of insertion. This suggests that one potential mechanism of type 2 LOS is due to injury to the RLN within the intralaryngeal component of the larynx that cannot be tested, rather than due to anatomical variations (such as bifurcations of the RLN at the inferior thyroid artery, sympathetic fascial bands, a small anterior motor branch of a RLN bifurcating near the Ligament of Berry, compression between fascial leaflets at the Ligament of Berry or global stretch injury) [5, 25, 26]. Other studies have found that rotational forces through the larynx and trachea cause higher tracheal tube cuff pressures and consequently lower electromyography amplitudes, leading to diffuse type 2 LOS [27, 28].

In addition, we did not find any association on univariate regression analysis between patient variables of age, sex, BMI, largest nodule, preoperative ultrasound lobe volume, central lymph node dissection or indication for surgery as being predictive for loss of signal (Table 5). Longer operative duration and male sex have been associated with increased RLN palsy rates; however, intralaryngeal factors such as endotracheal tube size, intubation grade, Mallampati score and BMI did not increase RLN palsy rates or risk of focal nerve injury in a large retrospective study [29]. Female sex has also been found to be weakly associated with transient RLN palsy [30].

We reduced confounding by neuromuscular blockade by reversing all patients immediately after intubation. This is consistent with our finding of no significant difference in EMG amplitude between left and right baseline vagus stimulation (V1) in patients undergoing total thyroidectomy (*P* = 0.3942). However, limitations of the present study include potential positional changes of the endotracheal tube during the procedure. Rotational and depth changes of the endotracheal tube are documented to cause EMG amplitude changes [31–33]. We also attempted to reduce bias with a standardised surgical approach by a single surgeon, utilising the same nerve monitoring system, and minimising EMG confounding variables by using a single anaesthetist with placement of the tube under direct vision. Additionally, selection bias was reduced by selecting consecutive thyroidectomy cases.

Further studies could include a clinical trial in electromyography comparing EMG amplitude changes in a retrograde RLN dissection, compared to the lateral approach utilised in the current study, which could potentially identify an alternative method in reducing traction forces on the RLN.

## Conclusion

A significant decrease in vagus nerve EMG amplitude occurs during medialisation of the thyroid lobe in conventional thyroidectomy. Stretch injury or compression during thyroid mobilisation, most likely between the vagus and RLN stimulation sites, and within the intralaryngeal component of the RLN, is the most likely cause of RLN impairment during thyroidectomy.

## References

[1] Chiang FY, Lu IC, Tsai CJ (2011). Does extensive dissection of recurrent laryngeal nerve during thyroid operation increase the risk of nerve injury? Evidence from the application of intraoperative neuromonitoring. Am J Otolaryngol.

[2] Christou N, Mathonnet M (2013). Complications after total thyroidectomy. J Visc Surg.

[3] Calo PG, Medas F, Gordini L (2016). Interpretation of intraoperative recurrent laryngeal nerve monitoring signals: the importance of a correct standardization. Int J Surg.

[4] Anuwong A, Lavazza M, Kim HY (2016). Recurrent laryngeal nerve management in thyroid surgery: consequences of routine visualization, application of intermittent, standardized and continuous nerve monitoring. Updates Surg.

[5] Liddy W, Wu CW, Dionigi G (2021). Varied recurrent laryngeal nerve course is associated with increased risk of nerve dysfunction during thyroidectomy: results of the surgical anatomy of the recurrent laryngeal nerve in thyroid surgery study, an international multicenter prospective anatomic and electrophysiologic study of 1000 monitored nerves at risk from the international neural monitoring study group. Thyroid.

[6] Schneider R, Randolph GW, Dionigi G (2018). International neural monitoring study group guideline 2018 part I: staging bilateral thyroid surgery with monitoring loss of signal. Laryngoscope.

[7] Gur EO, Haciyanli M, Karaisli S (2019). Intraoperative nerve monitoring during thyroidectomy: evaluation of signal loss, prognostic value and surgical strategy. Ann R Coll Surg Engl.

[8] Francis DO, Pearce EC, Ni S (2014). Epidemiology of vocal fold paralyses after total thyroidectomy for well-differentiated thyroid cancer in a medicare population. Otolaryngol Head Neck Surg.

[9] Jeannon JP, Orabi AA, Bruch GA (2009). Diagnosis of recurrent laryngeal nerve palsy after thyroidectomy: a systematic review. Int J Clin Pract.

[10] Yu Q, Liu K, Zhang S (2019). Application of continuous and intermittent intraoperative nerve monitoring in thyroid surgery. J Surg Res.

[11] Genther DJ, Kandil EH, Noureldine SI (2014). Correlation of final evoked potential amplitudes on intraoperative electromyography of the recurrent laryngeal nerve with immediate postoperative vocal fold function after thyroid and parathyroid surgery. JAMA Otolaryngol Head Neck Surg.

[12] Chiang FY, Lu IC, Kuo WR (2008). The mechanism of recurrent laryngeal nerve injury during thyroid surgery–the application of intraoperative neuromonitoring. Surgery.

[13] Schneider R, Przybyl J, Pliquett U (2010). A new vagal anchor electrode for real-time monitoring of the recurrent laryngeal nerve. Am J Surg.

[14] Omura T, Sano M, Omura K (2004). A mild acute compression induces neurapraxia in rat sciatic nerve. Int J Neurosci.

[15] Schneider R, Randolph G, Dionigi G (2016). Prospective study of vocal fold function after loss of the neuromonitoring signal in thyroid surgery: the international neural monitoring study group’s POLT study. Laryngoscope.

[16] Dionigi G, Wu CW, Kim HY (2016). Severity of recurrent laryngeal nerve injuries in thyroid surgery. World J Surg.

[17] Serpell JW (2010). New operative surgical concept of two fascial layers enveloping the recurrent laryngeal nerve. Ann Surg Oncol.

[18] Serpell JW, Lee JC, Yeung MJ (2014). Differential recurrent laryngeal nerve palsy rates after thyroidectomy. Surgery.

[19] Serpell JW, Lee JC, Chiu WK (2015). Stressing the recurrent laryngeal nerve during thyroidectomy. ANZ J Surg.

[20] Schneider R, Bures C, Lorenz K (2013). Evolution of nerve injury with unexpected EMG signal recovery in thyroid surgery using continuous intraoperative neuromonitoring. World J Surg.

[21] Byun SH, Ahn KM (2021). Functional and electron-microscopic changes after differential traction injury in the sciatic nerve of a rat. Maxillofac Plast Reconstr Surg.

[22] Phelan E, Schneider R, Lorenz K (2014). Continuous vagal IONM prevents recurrent laryngeal nerve paralysis by revealing initial EMG changes of impending neuropraxic injury: a prospective, multicenter study. Laryngoscope.

[23] Schneider R, Randolph GW, Sekulla C (2013). Continuous intraoperative vagus nerve stimulation for identification of imminent recurrent laryngeal nerve injury. Head Neck.

[24] Chiang FY, Lee KW, Chen HC (2010). Standardization of intraoperative neuromonitoring of recurrent laryngeal nerve in thyroid operation. World J Surg.

[25] Snyder SK, Lairmore TC, Hendricks JC (2008). Elucidating mechanisms of recurrent laryngeal nerve injury during thyroidectomy and parathyroidectomy. J Am Coll Surg.

[26] Papachristos AJ, Sidhu SB (2021). Thyroidectomy technique: defusing the recurrent laryngeal nerve - the superior approach. Am J Surg.

[27] Randolph GW, Dralle H, International Intraoperative Monitoring Study (2011). Electrophysiologic recurrent laryngeal nerve monitoring during thyroid and parathyroid surgery: international standards guideline statement. Laryngoscope.

[28] Taylor JW, Soeyland K, Ball C (2020). Changes in tracheal tube cuff pressure and recurrent laryngeal nerve conductivity during thyroid surgery. World J Surg.

[29] Moreira A, Forrest E, Lee JC (2020). Investigation of recurrent laryngeal palsy rates for potential associations during thyroidectomy. ANZ J Surg.

[30] Thomusch O, Machens A, Sekulla C (2000). Multivariate analysis of risk factors for postoperative complications in benign goiter surgery: prospective multicenter study in Germany. World J Surg.

[31] Liu XL, Wu CW, Zhao YS (2016). Exclusive real-time monitoring during recurrent laryngeal nerve dissection in conventional monitored thyroidectomy. Kaohsiung J Med Sci.

[32] Tsai CJ, Tseng KY, Wang FY (2011). Electromyographic endotracheal tube placement during thyroid surgery in neuromonitoring of recurrent laryngeal nerve. Kaohsiung J Med Sci.

[33] Kim HY, Tufano RP, Randolph G (2016). Impact of positional changes in neural monitoring endotracheal tube on amplitude and latency of electromyographic response in monitored thyroid surgery: results from the porcine experiment. Head Neck.

